# Isolation of a novel species in the genus *Cupriavidus* from a patient with sepsis using whole genome sequencing

**DOI:** 10.1371/journal.pone.0232850

**Published:** 2020-05-13

**Authors:** Oh Joo Kweon, Yong Kwan Lim, Hye Ryoun Kim, Tae-Hyoung Kim, Sung-min Ha, Mi-Kyung Lee

**Affiliations:** 1 Department of Laboratory Medicine, Chung-Ang University College of Medicine, Seoul, Republic of Korea; 2 Department of Urology, Chung-Ang University College of Medicine, Seoul, Republic of Korea; 3 ChunLab, Inc., Seoul, Republic of Korea; Institut National de la Recherche Agronomique, FRANCE

## Abstract

Whole genome sequencing (WGS) has become an accessible tool in clinical microbiology, and it allowed us to identify a novel *Cupriavidus* species. We isolated Gram-negative bacillus from the blood of an immunocompromised patient, and phenotypical and molecular identifications were performed. Phenotypic identification discrepancies were noted between the Vitek 2 (bioMérieux, Marcy-l’Étoile, France) and Vitek MS systems (bioMérieux). Using 16S rRNA gene sequencing, it was impossible to identify the pathogen to the species levels. WGS was performed using the Illumina MiSeq platform (Illumina, San Diego, CA), and genomic sequence database searching with a TrueBac^TM^ ID-Genome system (ChunLab, Inc., Seoul, Republic of Korea) showed no strains with average nucleotide identity values higher than 95.0%, which is the cut-off for species-level identification. Phylogenetic analysis indicated that the bacteria was a new *Cupriavidus* species that formed a subcluster with *Cupriavidus gilardii*. WGS holds great promise for accurate molecular identification beyond 16S rRNA gene sequencing in clinical microbiology.

## Introduction

The genus *Cupriavidus* belongs to the Gram-negative β–proteobacteria that have been found in human clinical specimens and environmental sources [1, 2]. The genus *Cupriavidus* was first proposed in 1987 by Makkar and Casida, who described *Cupriavidus necator*: a nonobligate bacterial predator of bacteria in soil [3, 4]. Since then, a total of 17 recognized *Cupriavidus* species have been reported [5], and to date, *C*. *gilardii* [1, 6, 7], *C*. *metallidurans* [8], and *C*. *pauculus* [9, 10] have been isolated from human clinical specimens.

In clinical microbiology, 16S ribosomal RNA (16S rRNA) gene sequencing has been widely used for the molecular identification of isolates when conventional phenotypic methods have failed to produce the correct identification. This is because the 16S rRNA gene is a phylogenetic marker that plays an essential role in the development of bacterial phylogeny and classifications [11, 12]. However, in many cases, some species share a high level of 16S rRNA gene sequence similarity (99%), which makes it difficult to differentiate two species using 16S rRNA sequencing alone [12, 13].

Whole genome sequencing (WGS) is now being used more widely due to the introduction of cost-effective, high-throughput, next-generation sequencing. Unlike 16S rRNA gene sequencing, WGS provides clear-cut criteria for bacterial classification usint the average nucleotide identity (ANI) value [11, 12]. The ANI represents a mean of similarity values between homologous genomic regions shared by two genes. It is the most widely used for genomic sequence similarity and is regarded as a possible next-generation gold standard for species delineation, providing an alternative to DNA-DNA hybridization values. It is now generally accepted that ANI values of 95% to 96% equate to a DNA–DNA hybridization value of 70%, which is the accepted standard for bacterial species demarcation [14].

In the present study, we investigated a case of bloodstream infection caused by Gram-negative rods that were determined using WGS to be a novel *Cupriavidus* species. The database search and phylogenetic tree analysis were conducted using genomic sequencing, and the characteristics of the novel species, including its phenotype and antimicrobial susceptibility, were described.

## Materials and methods

### Ethics statement

Because the patient was expired before the time of bacteria identification, Institutional Review Board (IRB) review of the study and the need for obtaining informed consent from the patient for the publication were waived according to the Chung-Ang University Hospital IRB policy (IRB No. 2001-001-19296). The official report of IRB review which contains the approval for exemption from IRB review was uploaded.

### Clinical case

A 26-year-old woman was admitted to Chung-Ang University Hospital for induction chemotherapy with idarubicin and cytarabine for pre-diagnosed acute myeloid leukemia with myelodysplasia-related changes. On the first day of chemotherapy (D0), neutropenic fever developed (body temperature of 38°C, absolute neutrophil count of 185/μL). Without identifying the exact origin of the fever, piperacillin/tazobactam (IV, 4.5g, q6hr) were administered as empirical antibiotics. Despite treatment, the fever did not subside. On D18, vancomycin-resistant Enterococci (VRE) were found in urine culture; antibiotic treatment was changed to meropenem (IV, 2g, q8hr) and teicoplanin (IV, 0.4g, qp). VRE was found again in the patient’s urine on D27 as well as in blood culture, and the patient experienced abdominal pain, diarrhea, and sustained fever. Vancomycin (IV, 100ml, bid) was administered as an alternative to the teicoplanin for the management of neutropenic fever. The next day, abdominal pain and diarrhea subsided, however, the fever did not subside despite the serial administration of antibiotics. On D31, blood cultures obtained from five different sites (two different sites of peripheral venipuncture and three lumens of Hickman lines) were positive for Gram-negative rods from direct Gram stains, and VRE ceased growing. Identical pathogens were also solely grown in the blood obtained from the two lumens of the dual lumen catheter and one central venous line on D33. On D33, the patient died from septic shock and related multiple organ failure.

### Initial laboratory assessment for the identification of the pathogen

In the blood culture samples obtained on D31 and D33, there were positive signals in aerobic bottles (BacT/Alert FA Plus, bioMérieux, Inc., Marcy-l’Étoile, France) after roughly 13 to 15 hours of incubation at 35.0°C using the BacTAlert 3D blood culture system (bioMérieux, Inc.). About 1 mL of blood with a culture broth from the positive bottles was inoculated in blood agar plates and MacConkey agar plates and incubated at 37°C with a 5% CO_2_ supplement condition. With the colonies on blood agar plates (BAP), Gram staining was conducted and automated phenotypical identification systems including Vitek 2 (bioMérieux, Inc.) and Vitek MS v.2.0 systems (bioMérieux, Inc.) of MALDI-TOF analyzers were used. 16S rRNA gene sequencing analyses with the universal primers of 27F and 1492R were performed for a species-level identification according to the CLSI document MM18-A [15].

### Additional biochemical tests

Growth in R2A agar (Becton Dickinson, NJ, USA) was assessed at different temperatures (4, 10, 15, 20, 25, 30, 37, 40, and 45°C), at various pH values (pH 3–11 at intervals of 1.0 pH units), and with different NaCl concentrations (0, 0.5, 1–20% NaCl at intervals of 1.0%) after a 24hr incubation period. Catalase activity tests using 3% H_2_O_2_ solution and oxidase tests using oxidase reagent (bioMérieux, Inc.) were performed. To test the ability to ferment sugars and to produce hydrogen sulfide (H_2_S), bacteria were inoculated into a Triple Sugar Iron (TSI) agar slant. Other biochemical characteristics and enzyme activities were assessed using the Vitek 2 GN ID Card (bioMérieux, Inc.).

### Whole genome sequencing

Genomic DNA was prepared using the preferred methods or kits in accordance with the manufacturer’s instructions. Quantification of DNA concentration was done using Quat-iT PicoGreen dsDNA Assay kit (Thermo Fisher Scientific, Waltham, MA). The library was constructed using the TruSeq Nano DNA LT Library Prep kit (Illumina, San Diego, CA) according to the manufacturer’s guidelines. The library was quantified using the Bioanalyzer 2100 (Agilent Technologies, Santa Clara, CA) with the DNA 7500 kit (Agilent Technologies). WGS was performed on the Illumina MiSeq platform 2x300bp (Illumina, San Diego, CA). The raw data from the MiSeq instrument in the FASTQ format were directly uploaded and analyzed with the TrueBac^TM^ ID-Genome system (www.truebacid.com, ChunLab, Inc., Seoul, Republic of Korea). The TrueBac^TM^ ID-Genome system was operated along with the workflow suggested by Chun et al.[16]; it found the phylogenetic neighbors using the Mash program (*in silico* method for genome distance estimation tool) [17] and sequences of *16S rRNA*, *recA*, and *rplC* extracted from the genomic data, and then it calculated the ANI values compared to the neighbor species using genomic sequencing with the MUMmer tool [11]. For genome-based classification at the species level, cut-off values for identity percentages for 16S rRNA sequences and ANI values for whole genome sequencing were designated as 98.7% and 95.0%, respectively, according to the proposed minimal standards for the use of genome data for the taxonomy of prokaryotes [16, 18]. In addition to bacterial identification, the antimicrobial resistance gene profile was obtained using the Comprehensive Antibiotic Resistance Database search [19]. Possible contamination of genomic data by other organisms was screened using the ContEst16S method (ChunLab, Inc.), in which 16S rRNA gene fragments are screened to determine whether the genome assembly is contaminated [20].

### Phylogenetic analysis

Phylogenetic analyses were conducted with both 16S rRNA sequencing and WGS. With 16S rRNA gene sequencing, the phylogenetic tree was constructed using the Neighbor-Joining method with MEGA X (Molecular Evolutionary Genetic Analysis, version 10.1) [21–23]. The stability of the grouping was estimated by the bootstrap test (1000 replicates) [24]. With the whole genome sequence, the genome tree was generated using the unweighted pair group method with arithmetic mean clustering method and the ANI value [25]. The complementary ANI value to 1 was used to calculate the distance. Pairwise ANI of each genome was calculated using OrthoANI [26].

### Data availability and strain deposition

The 16S rRNA sequence of the isolated bacteria was uploaded on the GenBank database with an accession No. MN453488 (https://www.ncbi.nlm.nih.gov/nuccore/MN453488). The whole genome sequence of the isolated bacteria was uploaded to the NCBI Assembly Database with GenBank Assembly Accession No. GCA_008632125.1 and RefSeq Assembly Accession No. GCF_008632125.1 (https://www.ncbi.nlm.nih.gov/assembly/GCA_008632125.1). The strain was deposited in NCCP, the National Culture Collection for Pathogens, operated by the Korean CDC (http://nccp.cdc.go.kr), with the Assession No. of NCCP 16966.

### Antimicrobial susceptibility testing

For the antimicrobial susceptibility testing (AST), the Vitek2 AST-N224, AST-P600 (for linezolid only) (bioMérieux, Inc.) and disk diffusion tests were used. Antimicrobial agents, disk contents, and zone diameter breakpoints for disk diffusion, and MIC breakpoints for Vitek2 AST-N224 were guided by the Clinical and Laboratory Standards Institute (CLSI) document M100 [27] for *Acinetobacter baumannii*, the representative microorganism of Gram-negative rods. For the disk diffusion tests, a 0.5 McFarland standard value of the isolate was inoculated onto Mueller-Hinton agar and BBL^TM^ Sensi-Disc^TM^ Antimicrobial Susceptibility Test Discs (BD Biosciences, Franklin Lakes, NJ) were used for piperacillin (100 μg), piperacillin/tazobactam (100/100 μg), ticarcillin/clavulanate (75/10 μg), ceftazidime (30 μg), cefepime (30 μg), ceftriaxone (30 μg), imipenem (10 μg), meropenem (10 μg), colistin (10 μg), gentamicin (10 μg), tetracycline (30 μg), ciprofloxacin (5 μg), levofloxacin (5 μg), and trimethoprim/sulfamethoxazole (1.12/23.75 μg). The agar plate was incubated for 18 hours in 35°C and 5% CO_2_ conditions. The zone diameter was read and interpreted. For Colistin, broth microdilution (BMD) was additionally performed to determine MIC accurately, in accordance with guidelines of the joint CLSI-EUCAST Polymyxin Breakpoints Working Group [28].

## Results

### Conventional phenotypic and molecular pathogen identification

Colonies on blood agar plates were gray, translucent, and 1mm in size. A Gram stain showed Gram-negative rods, and the bacillus was oxidase-negative and catalase-positive. Subcultures on a MacConkey agar plate revealed that the bacillus was a non-lactose fermenter, and on a triple sugar iron agar, it showed a K/A pattern, and was negative for H_2_S and gas formation. In R2A agar, growth occurred at 30-45°C, with an optimal temperature of 40°C. The pH range for growth was 6.0–10.0 (optimal pH: 7.0), and growth occurred with 0–10% of NaCl (optimal NaCl: 0.5%) after 24hrs of incubation. The bacillus was positive for L-proline Arylamidase, phosphatase, L-lactate alkalinization, and succinate alkalinization. Acid was not produced from L-arabitol, D-glucose, D-maltose, D-mannitol, D-mannose, D-sorbitol, adonitol, and was negative for lipase, phosphatase, urease, β-glucosidase, β-xylosidase, β-N-acetyl-glucosaminidase, glutamyl arylamidase pNA, β-alanine arylamidase pNA, L-proline arylamidase, tyrosine arylamidase, β-N-acetylgalactosaminidase, β-glucuronidase, α-galactosidase, ornithine decarboxylase, lysine decarboxylase, L-histidine assimilation, L-malate assimilation, L-lactate assimilation, and malonate.

Vitek 2 (bioMérieux, Inc.) identified the microorganism as *Bordetella hinzii* with an identification probability of 99%, and the Vitek MS system revealed that this pathogen was *C*. *gilardii* with a confidence value of 99.9%. 16s rRNA gene sequencing in conjunction with GenBank BLAST search identified the pathogen as *C*. *plantarum* with the highest value of identification percentage of 99.32% (1467/1477 bp) followed by *C*. *taiwanensis* (98.98%) and *C*. *metallidurans* (98.98%). Although the highest identification percentage was higher than 99.0%, because it was not greater than 0.8% separation between different species, it was impossible to identify the pathogen to the species levels according to the CLSI guidelines [15].

### Whole genome sequencing

The genome size of the pathogen obtained from WGS was 5,750,268 bp. GC contents were 67.87%, average sequencing depth was 294.57, the total number of contigs was 76, and N50 value was 163,605 bp. There was only one 16S rRNA gene sequence in the genomic sequence; thus, we concluded that the genome assembly was not contaminated by other organisms. From the TrueBac^TM^ ID Database search, seven *Cupriavidus* species showed a higher 16S rRNA gene identity percentage of more than 98.7%, but we did not find any pathogen with ANI values higher than 95.0%, which is the algorithmic cut-off for species-level identification. *C*. *gilardii* showed the highest ANI value of 92.07% (ANI coverage of 84.4%) with 16S rRNA gene (obtained from the whole genome sequence) identity of 99.72%. *C*. *plantarum* showed the second highest 16s rRNA sequence identity (99.24%) which was obtained from the whole genome sequence, but ANI was only 85.58%. Details of the database search to identify the pathogen are listed in Table 1, and Mash results of genomic sequence are listed in S1 Table.

**Table 1:** The results of whole genome sequence database (the TrueBac^TM^ ID-Genome system) matching for the novel *Cupriavidus* species.

	Species (Hit taxon)	ANI (%)	ANI coverage (%)	16S rRNA gene identity (%)^a^
1	*Cupriavidus gilardii*	92.07	84.4	99.72
2	*Cupriavidus plantarum*	85.58	23.7	99.24
3	*Cupriavidus taiwanensis*	86.45	35.8	98.97
4	*Cupriavidus pauculus*	85.80	26.0	98.97
5	*Cupriavidus metallidurans*	85.69	22.9	98.97
6	*Cupriavidus nantongensis*	87.15	37.2	98.76

Abbreviation: ANI, average nucleotide identity.

^a^ Obtained from the whole genome sequence

From the Comprehensive Antibiotic Resistance Database search, the pathogen was revealed to have *MCR-5* gene, which is known to be associated with Colistin resistance of *Enterobacteriales* with a 100% identity percentage, bit score/cutoff of 1127.08/1000, and a decision level considered “perfect.” The genomic sequence also showed a 100% identity match to the *mdtE* gene; however, it had a lower bitscore value than the cutoff (21.17/675) and was therefore classified as a “strict” decision level.

### Phylogenetic tree analysis

**Fig 1:**
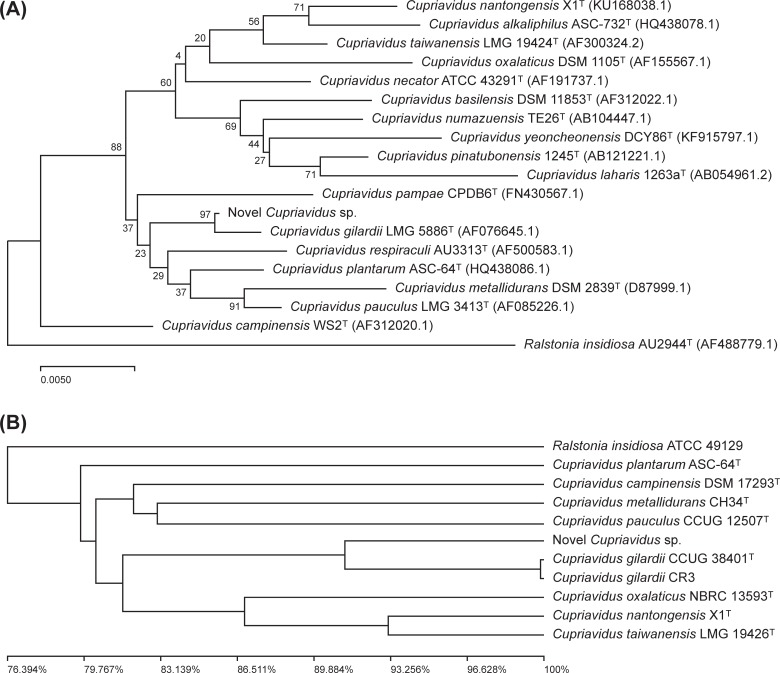
Phylogenetic tree based on 16S rRNA gene sequencing and whole genome sequencing in the relationships of novel *Cupriavidus* species and the other species of the genera *Cupriavidus* and *Ralstonia*. A) Neighbor-joining phylogenetic trees based on 16S rRNA gene sequences using *Ralstonia insidiosa* as an outgroup. The percentage of replicated trees in which the associated taxa clustered together in the bootstrap test (1000 replicates) are shown next to the branches. The evolutionary distances were computed using the Jukes-Cantor method. B) The genomic phylogenetic tree generated by the unweighted pair group method with arithmetic mean clustering method and average nucleotide identity value using *Ralstonia insidiosa* as an outgroup. The complementary average nucleotide identity value to 1 was used to calculate the distance. The tree was drawn to scale, with branch lengths in the same units as those of the evolutionary distances used to infer the phylogenetic tree.

Phylogenetic tree analysis using the Neighbor-Joining method with 16s rRNA gene sequencing revealed that this newly isolated species formed a subcluster with *Cupriavidus gilardii* LMG 5886^T^ with 97% bootstrap support (Fig 1A). A similar subcluster was found in phylogenetic tree analysis using WGS generated by the unweighted pair group method with arithmetic mean clustering method and ANI values. The target pathogen had the closest relationship with *Cupriavidus gilardii* CCUG 38401^T^ and *C*. *gilardii* CR3, but it is a distinctive species distinguishable from *C*. *gilardii* (Fig 1B).

### Antimicrobial susceptibility test

The results of AST performed with Vitek2 AST-N224, AST-P600 (for linezolid only) and disk diffusion tests are listed in Table 2. From the Vitek2 AST-N224, the novel *Cupriavidus* species showed “resistance” to the agents of piperacillin/tazobactam, ceftazidime, meropenem, and gentamicin. In the disk diffusion tests, meropenem and gentamicin disks showed resistance results. However, piperacillin/tazobactam, and ceftazidime showed susceptibility and intermediate results, respectively. For colistin, not only both Vitek2 AST-N224 and disk diffusion tests showed susceptible results, but the BMD tests showed the same. BMD tests showed a MIC of 0.5 μg/mL, and it was the same results to the Vitek2 AST-N224.

**Table 2:** Antimicrobial susceptibility profiles including minimum inhibitory concentrations from Vitek2 AST-N224. AST-P600 (bioMérieux, Inc.) and zone diameter from disk diffusion tests after 16 hours incubation on Mueller-Hinton agar of the novel *Cupriavidus* species.

Antimicrobial Agent	MIC^a^ (μg/mL)	Zone Diameter (mm)	Antimicrobial Agent	MIC^a^ (μg/mL)	Zone Diameter (mm)
**Ampicillin /Sulbactam**	≤2 (S)^b^	Not tested	**Aztreonam**	≥64 (R)	Not tested
**Ticarcillin /Clavulanic Acid**	≤8 (S)	15 (I, 75/10 μg)^b^	**Colistin**	≤0.5^c^ (S)	20 (S, 10 μg)
**Piperacillin**	64 (I)	30 (S, 100 μg)	**Gentamicin**	≥16 (R)	0 (R, 10 μg)
**Piperacillin /Tazobactam**	≥128 (R)	40 (S, 100/10 μg)	**Tetracycline**	Not tested	40 (S, 30 μg)
**Cefotaxime**	2 (S)	Not tested	**Ciprofloxacin**	0.5 (S)	35 (S, 5 μg)
**Ceftazidime**	≥64 (R)	15 (I, 30 μg)	**Levofloxacin**	Not tested	35 (S, 5 μg)
**Cefepime**	≤1 (S)	35 (S, 30 μg)	**Trimethoprim-/sulfamethoxazole**	≤20 (S)	40 (S, 1.25/23.75 μg)
**Ceftriaxone**	Not tested	40 (S, 30 μg)	**Amikacin**	16(S)	Not tested
**Imipenem**	1 (S)	30 (S, 10 μg)	**Minocycline**	2 (S)	Not tested
**Meropenem**	≥16 (R)	0 (R, 10 μg)	**Tigecycline**	≤0.5 (S)	Not tested
**Linezolid**	≥8^d^	Not tested			

Abbreviation: MIC, minimum inhibitory concentration

^a^ Determined by Vitek2 AST-N224 or AST-P600 (for linezolid only) (bioMérieux, Inc.).

^b^ Interpreted according to the breakpoints listed in “Performance Standards for Antimicrobial Disk Susceptibility Tests. 13th Ed. CLSI standard M100” for *Acinetobacter* spp.

^c^ An identical result was also obtained from broth microdilution test (BMD) which was performed according to the CLSI-EUCAST Polymyxin Breakpoints Working Group

^d^ Susceptibility was not determined due to the lack of breakpoint data for Gram-negative rods.

## Discussion

The genus *Cupriavidus* has a complex history regarding their taxonomy [4]. The genera *Cupriavidus* and *Ralstonia* belong to the family *Burkholderiaceae* in the class *Burkholderiales*. The species of the genus *Ralstonia* had been divided into two distinct genera: *Ralstonia* and the new genus *Wautersia* based on the 16S rRNA gene sequence analysis in 2004 [29]. Further study revealed that *Wautersia eutropha*, the type species of the genus *Wautersia*, was an identical strain of *C*. *necator*, the type species of the genus *Cupriavidus*, and all species of the genus *Wautersia* were transferred to the *Cupriavidus* [4].

Although the pathogenicity of the genus *Cupriavidus* in human infection is unclear, the evidence of its pathogenicity are growing especially for *C*. *gilardii*, the most closely related species to the novel *Cupriavidus* species isolated in this study. *C*. *gilardii* was isolated from the blood, throat, abscess, or stool specimens in immunocompromised patients [1, 6, 7]. Most patients respond well to antibiotics, but a fatal outcome from septic conditions has also been reported in a child with aplastic anemia [6]. Bloodstream infection due to *C*. *gilardii* has also been reported not only in patients with immunosuppression, but also in patient without obvious immunodeficiency [1]. In the present study, although the patient had been suffering from VRE, which was finally isolated at D27, a novel *Cupriavidus* species was solely isolated from blood culture obtained at D31 from five different sites (two peripheral venipuncture, Hickman lines, dual lumen catheter, and central venous line) in the immunocompromised patient, resulting in a fatal outcome due to sepsis-related multi-organ failure at D33. Thus, the novel *Cupriavidus* species should be considered a pathogen or a cause of human infection.

Whole genome sequencing has considerable potential in clinical microbiology, as it could provide accurate identification and facilitate decisions regarding the recognition of novel species [14]. To replace the DNA–DNA hybridization, several values were developed to reflect similarity between two genome sequences. Such values were coined as the *overall genome relatedness index* (OGRI) [16], and among the OGRI, ANI has been most widely used and in general, 95.0% ANI is used as the cut-off value for species-level identification [14]. The utility of genome-based prokaryote identification in clinical microbiology greatly depends on the quality or the volume of the database. The TrueBac^TM^ ID-Genome system was the first commercial whole genome-based bacterial identification system, and its database contains highly curated and taxonomically validated genome data with type and reference strains [11]. We used the database version 20190524 which contains 12,419 genomes representing 12,108 species and 311 subspecies. The database also contains 20,007 16S rRNA gene sequences. In the present study, there were no strains with higher ANI values than 95.0% in the TrueBac^TM^ ID-Genome system; therefore, we concluded that the pathogen is a novel species in the genus *Cupriavidus*.

In clinical microbiology, automated phenotypical identification systems and MALDI-TOF MS have been widely adopted for routine bacterial identification. However, discrepancies between methods are frequently encountered in routine practice, even if the identification score is high enough to identify species levels. This problem may originate from the overlapped biochemical or proteomic profiles of the isolates among the different strains, the limited number of databases/libraries in the analyzer, or both. Likewise, higher 16S rRNA gene sequence similarity compared to cut-off levels (98.7~99.0%) did not guarantee the correct identification of some strains, as almost identical 16S rRNA gene sequences have been reported in different species [12, 13]. These difficulties in accurately identifying species in either molecular or phenotypic typing can occur more frequently especially in the strains rarely found in clinical specimens, as in our case. The novel *Cupriavidus* in the study was originally identified as *B*. *hinzii* with an identification probability of 99% in Vitek2, as *C*. *gilardii* with a 99.9% of confidence value in the Vitek MS system, and as the genus *Cupriavidus*, most closely related to *C*. *plantarum* with a 99.32% identification percentage in 16S rRNA gene sequencing. In the isolates that show discrepant results according to the identification methods, WGS can be a helpful tool to achieve accurate species-level identification, or even to confirm the novel species.

In AST, piperacillin/tazobactam, ceftazidime, meropenem, and gentamicin showed resistance to Vitek2 AST-N224 or disk diffusion tests. In the case of *C*. *gilardii*, the species most closely related to the novel *Cupriavidus* species, several reports have been published regarding multi-drug resistance, including the aforementioned drugs, which reported that their patients had improved after treatment with ciprofloxacin, piperacillin/tazobactam, or tetracycline [1, 6, 7, 30, 31]. However, the optimal therapeutic regimen for treating *C*. *gilardii* infection remains unclear due to limited data and the availability of *C*. *gilardii* in acquiring resistance to antibiotics upon their continuous administration [6, 30]. In the present case, the patient was treated with linezolid and meropenem to counteract the VRE isolated from previous blood cultures. At the time, the newly isolated pathogen was revealed to be Gram-negative rods with multi-drug resistance, especially to meropenem. Additionally, linezolid was known to be ineffective against Gram-negative bacteria [32, 33]. The patient unfortunately expired, and considering the AST profile of the novel *Cupriavidus* species, third-generation cephalosporins such as cefotaxime, imipenem, tetracycline, or ciprofloxacin would had been more effective antibiotics for treatment.

We found that *MCR-5* genes are encoded in the genomic sequence of the novel *Cupriavidus* species, which was known to be related to colistin-resistance. The products of *MCR-5* are phosphoethanolamine transferases, adding phosphoethanolamine to the lipid A moiety of LPS, leading to a more cationic LPS structure and consequently resistance to colistin [34]. However, the novel *Cupriavidus* species in the present case did not show resistance to colistin in both BMD tests and disk diffusion tests. The mechanisms and frequencies of the genotype-phenotype discrepancy between *MCR-5* gene and colistin are questionable. Although studies of the discrepancies between phenotypic and genotypic antibiotics resistance profiles have been reported [35, 36], reports focusing on the *MCR-5* gene and colistin resistance are lacking in the literature. Further investigation focusing on *MCR-5* gene and colistin resistance in non-*Enterobacteriales* Gram-negative rods need to be performed.

In conclusion, we have described the sepsis case caused by Gram-negative rods confidently recognized as a novel *Cupriavidus* species using WGS. The novel *Cupriavidus* species was shown to be most closely related to *C*. *gilardii*. Genome-based identification can be a useful tool for clinical microbiology as the cost of WGS continues to decrease and the coverage of the database expands.

## Supporting information

S1 Table Results of Mash, the *in silico* method for estimating genome distance, for genomic sequencing of the novel *Cupriavidus* species. (PDF)
