# Supplementary material for: Isolation of a novel species in the genus Cupriavidus from a patient with sepsis using whole genome sequencing
Source: PLoS One. 2020 May 13;15(5):e0232850. doi: 10.1371/journal.pone.0232850 (PMC7219751; doi:10.1371/journal.pone.0232850)
Supplement: S1 Table — (PDF) [file pone.0232850.s001.pdf]

S1 Table. Results of Mash, the *in silico* method for estimating genome distance, for genomic sequencing of the novel *Cupriavidus* species.

Identity	Shared-hashes	Median-multiplicity	P-value	Accession No.	Taxon name
0.979767	651/1000	1	0	GCA_001281465.1	*Cupriavidus gilardii*
0.882821	73/1000	1	2.59E-318	GCA_900249755.1	*Cupriavidus taiwanensis*
0.873204	58/1000	1	2.74E-247	GCA_001598055.1	*Cupriavidus oxalaticus*
0.871746	56/1000	1	6.06E-238	GCA_000069785.1	*Cupriavidus taiwanensis*
0.860849	43/1000	1	4.68E-178	GCA_900249985.1	*Cupriavidus taiwanensis*
0.853596	36/1000	1	1.63E-146	GCA_000876015.1	*Cupriavidus basilensis*
0.852452	35/1000	1	4.69E-142	GCA_003854935.1	*Cupriavidus pauculus*
0.851276	34/1000	1	1.31E-137	GCA_001592245.1	*Cupriavidus oxalaticus*
0.850067	33/1000	1	3.56E-133	GCA_004217045.1	*Cupriavidus* sp. ASC-9842
